# The mycoparasitic yeast Saccharomycopsis schoenii predates and kills multi-drug resistant *Candida auris*

**DOI:** 10.1038/s41598-018-33199-z

**Published:** 2018-10-08

**Authors:** Klara Junker, Gustavo Bravo Ruiz, Alexander Lorenz, Louise Walker, Neil A. R. Gow, Jürgen Wendland

**Affiliations:** 1 Carlsberg Research Laboratory, Yeast & Fermentation, DK-1799 Copenhagen V, Denmark; 2 The Institute of Medical Sciences (IMS), MRC Centre for Medical Mycology at the University of Aberdeen, School of Medicine, Medical Sciences & Nutrition, University of Aberdeen, Foresterhill, Aberdeen, AB25 2ZD United Kingdom; 3 Vrije Universiteit Brussel, Bioengineering Sciences, Functional Yeast Genomics, BE-1050 Brussels, Belgium

## Abstract

*Candida auris* has recently emerged as a multi-drug resistant fungal pathogen that poses a serious global health threat, especially for patients in hospital intensive care units (ICUs). *C. auris* can colonize human skin and can spread by physical contact or contaminated surfaces and equipment. Here, we show that the mycoparasitic yeast *Saccharomycopsis schoenii* efficiently kills both sensitive and multi-drug resistant isolates of *C. auris* belonging to the same clade, as well as clinical isolates of other pathogenic species of the *Candida* genus suggesting novel approaches for biocontrol.

## Introduction

*Candida auris* was first seen as an agent of human disease in 2009, when it had been isolated from the ear canal of a patient in Japan^1^. It has subsequently spread rapidly around the world and is now a major health threat on a global scale having been associated with potentially lethal infections in patients in ICUs in Eastern and South Asia, South Africa, Europe, the USA, and South America. Sequence-based analyses have grouped *C. auris* isolates from around the world into at least four different clades, represented by clonal populations^2^. More than 90% of isolates are fluconazole resistant and many isolates are cross-resistant to more than one of the three major classes of antifungals – azoles, echinocandins and polyenes^2^. A few strains of this fungus are resistant to all of the major classes of antifungals used by doctors to treat fungal infections. The species has a propensity to colonize skin, and it has proven to be difficult to eradicate from ICUs. Worldwide mortality rates for disease cases with *C. auris* infections of the bloodstream approach 50%. Concern about the emergence and spread of *C. auris* has resulted in alerts being posted by the CDC (Centers for Disease Control and Prevention, USA), the ECDC (European Centre for Disease Prevention and Control, Sweden), and PHE (Public Health England, UK)^3–5^.

*C. auris* colonizes the skin of patients and can be transmitted via contact with patients or contaminated hospital fixtures, which has already resulted in several health care associated outbreaks^6^. The horizontal transmission potential of *C. auris* demands strict decontamination methods and infection prevention protocols, since mortality rates in patients with systemic infections can be up to 50%^2,7,8^.

Necrotrophic mycoparasitism describes the ability of a fungal species to kill other fungi^9^. For example, filamentous fungi in the *Trichoderma* genus have been well characterized over the last decades and are used as biocontrol agents against fungal plant pathogens^10^. Mycoparasites typically exhibit wide host ranges and due to their general mode of action in killing their prey may use broad-acting lytic enzymes, such as proteases and chitinases. However, some species, e.g. *Trichoderma*, also generate specialized penetration structures or haustoria that allow them to efficiently target and kill prey cells^11,12^. Active mycoparasitism in yeast was discovered only in 1997, when species of the genus *Saccharomycopsis* were first described as necrotrophic predacious yeasts^13^. It has not been studied whether *Saccharomycopsis* species attack *Candida* species other than *Candida albicans*^13,14^. Predacious behaviour depends on solid structural support, presumably to allow for stable cell-cell contact and has been suggested to be starvation induced^14^. Specifically, a lack of organic sulfur-containing organic compounds such as methionine has been suggested as a trigger for predation, as all *Saccharomycopsis* yeasts share the, for microorganisms, rare feature of being unable to assimilate sulfate as their sole source of sulfur. Recently, we reported the lack of eight genes in the sulfate assimilation pathway in draft genomes of *Saccharomycopsis fodiens* and *Saccharomycopsis fermentans*^15,16^.

Here we show that *S. schoenii* efficiently attacks and kills a range of pathogenic *Candida* species, including the newly emerged human pathogenic fungus *C. auris*. We follow the predation process using time lapse microscopy in combination with fluorescent dyes. Efficient predation as shown here could be useful for biocontrol purposes in either clinical settings for skin clearance or in agricultural settings for combatting plant pathogens.

## Results and Discussion

**Figure 1:**
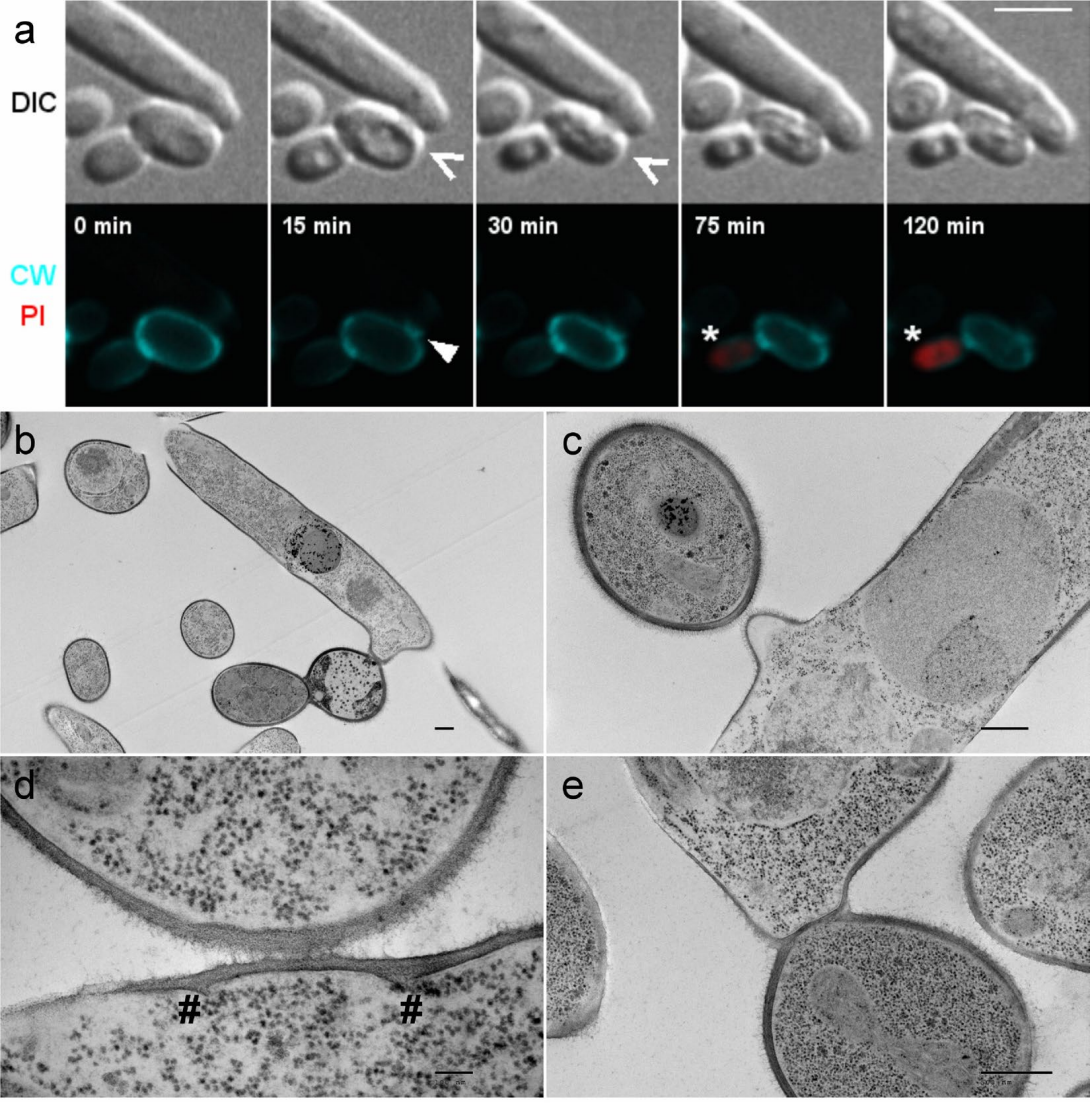
*S. schoenii* attacks and kills *C. auris*. (**a**) *S. schoenii* and *C. auris* NCPF8985#20 stained with Calcoflour White (CW, cyan, bottom panel), a fluorescent dye that stains chitin rich cell walls and septa, and ropidium iodide (PI, red, bottom panel), a fluorescent dye that stains nucleic acids of cells with a compromised cell membrane, i.e. dead or dying cells. We captured images twice per minute for two hours and found that at 15 min, a penetration peg [Δ] from *S. schoenii* is visualized by CW. The *C. auris* prey cell subsequently collapses in size between 15 and 30 min (Λ). Whereas the attacked *C. auris* cell was not stained by PI, its daughter cell accumulated PI between 75 min to 120 min (*). (**b**–**e**) TEM images of *S. schoenii* and *C. auris* that had been co-cultured for 1 h. Scale bar 500 nm in (**b**,**c** and **e**). Scale bar 100 nm in (**d**). (**b**) A dimorphic *S. schoenii* cell has formed a penetration peg to contact, attack and kill an ovoid *C. auris* cell**. (c**) A *S. schoenii* cell with a penetration peg protruding towards a prey cell. (**d**) Early interactions between *A. schoenii* and *C. auris* visualize potential penetration peg start sites (#). (**e**) Partial disintegration of the *C. auris* cell wall.

In this study, we prospected the use of a predatory yeast, *Saccharomycopsis schoenii*, as a potential biocontrol agent against human fungal pathogens of the *Candida* clade with a focus on *C. auris*. To this end we confronted multiple drug resistant strains including *C. auris* NCPF8985#20, a multi-drug resistant isolate from the South Asian clade (India), with *S. schoenii* (Supplementary Table 1). Equal numbers of dimorphic *S. schoenii* and ovoid *C. auris* NCPF8985#20 cells were seeded on minimal media agar on microscopy slides to offer solid support for a potential *S. schoenii* interaction. This minimal media lacked methionine and thus did not support proliferation of *S. schoenii* in pure culture. We found that *S. schoenii* attacked *C. auris* cells upon contact and killed prey cells using specialized penetration pegs (Fig. 1; Supplementary Movies 1–3). The chitin-staining fluorescent dye Calcofluor White, revealed septa at the bases of penetration pegs indicating the sites of entry (Fig. 1a; Supplementary Movies 1 and 2). Within minutes after *S. schoenii* cells generated penetration pegs, *C. auris* cells started to vacuolarize, take up dyes such as propidium iodide that are not permeable to living cells and then collapse, presumably because of feeding and material transfer to the predating *S. schoenii* cell (Fig. 1a; Supplementary Movies 1–3). We prepared Transmission Electron Microscopy (TEM) images of interactions between *S. schoenii* and *C. auris* after 1 h of co-culture on minimal media (Fig. 1b–e) and found that *C. auris* cells attacked by S*. schoenii* cells were necrotic (Fig. 1b). Penetration pegs were directed at prey cells (Fig. 1c) and cell wall interactions led to the formation of penetration peg start sites (# Fig. 1d). Ultimately this led to degradation of the prey cell wall (Fig. 1e). After killing of prey cells, penetration pegs did not grow further or develop into buds or daughter cells (Supplementary Movie 3). During their interaction with prey cells, *S. schoenii* cells did not show apical growth, however, *S. schoenii* cells resumed polar growth after successful attacks (Supplementary Movie 3).

**Figure 2:**
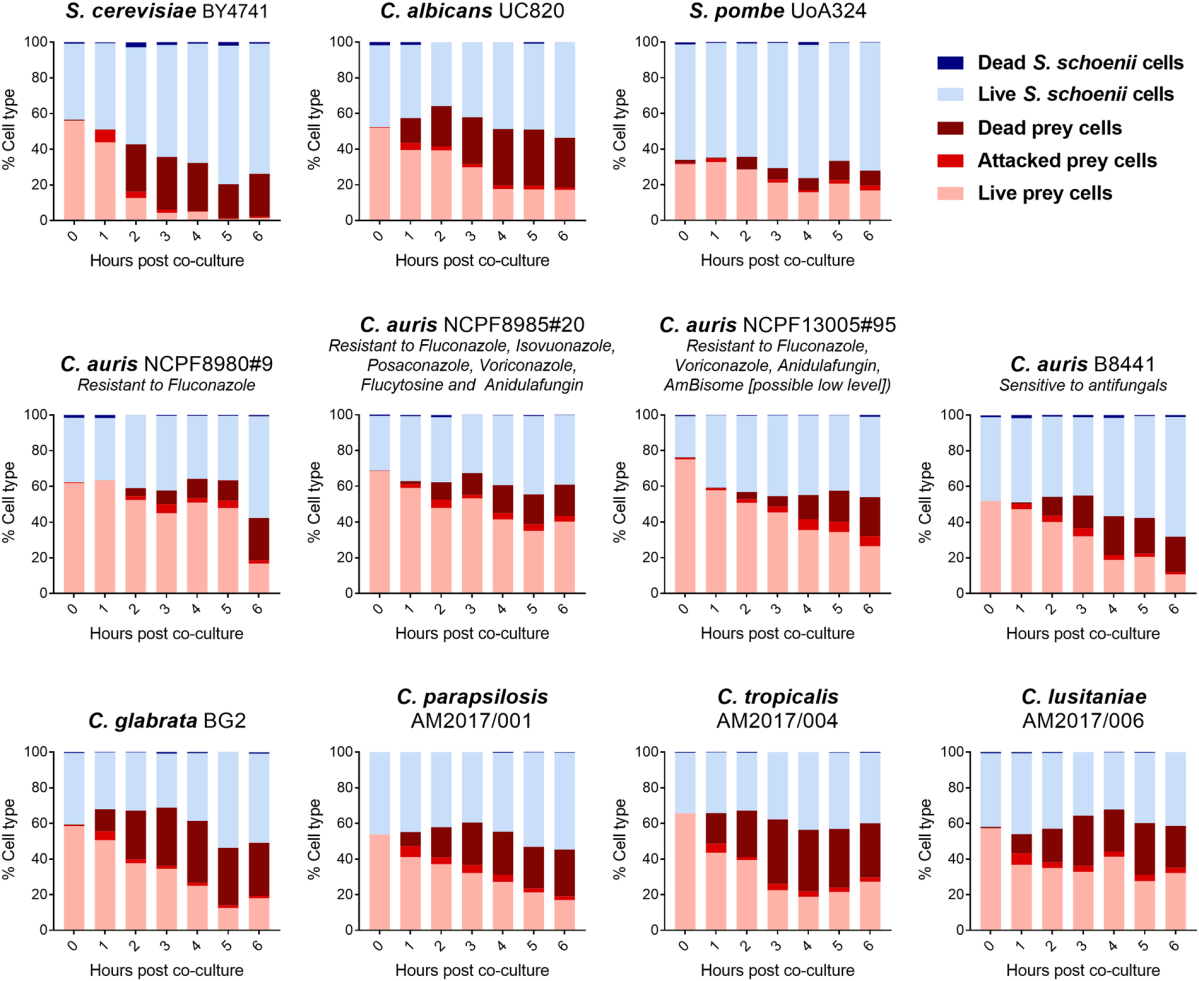
Kill curves of prey species attacked and killed by *S. schoenii*. Cells were co-cultured on several slides with SD media with 1% agarose, for up to 6 h. Every hour, we took one slide and captured 3 representative frames. Cells were scored on viability based on morphology and PI stain as per Fig. S2.

We determined rates of killing over a 6 h time-course using morphology and/or propidium iodide staining (PI) stain (Supplementary Figs 1 and 2). Several prey cells were found to accumulate PI staining upon predation, however, many prey cells were apparently killed without being stained by PI. In these cases, killed prey cells were “flattened” and or shrunken in size. This resulted in the death of around 34% of *C. auris* cells within a period of 6 h of co-culture with *S. schoenii* (Fig. 2, middle panel; Supplementary Table 2). As a control, almost none of the *C. auris* cells (0.6%) had died after 6 h when cultured alone under identical experimental conditions. To examine if predator-prey interactions differ with different *C. auris* isolates that exhibit variable drug resistance phenotypes, we analysed predator-prey interactions in three additional *C. auris* isolates (Supplementary Table 1). Furthermore, to elucidate host range of predator-prey interactions within *Candida* species we included clinical isolates of *Candida albicans*, *Candida glabrata, Candida lusitaniae, Candida parapsilosis* and *Candida tropicalis* in this analysis. For reference, we used *Saccharomyces cerevisiae* and *Schizosaccharomyces pombe*, two previously known prey species of *S. schoenii*^14^. All isolates of *Candida* species tested, including several drug resistant *C. auris* strains, were susceptible to predation by *S. schoenii* (Fig. 2 and Supplementary Table 2).

Collectively, these results demonstrate that the predator yeast *S. schoenii* provides a novel opportunity to develop biocontrol methods for skin disinfection. *Saccharomycopsis* predator are unique within the Saccharomycetes in displaying predatory behaviour. Thus these yeasts may harbor potential as biocontrol agents of other fungi including human and plant pathogens. Based on genome survey sequencing, *Saccharomycopsis* yeasts, like the distantly related filamentous ascomycete *Trichoderma*, harbor multi-gene families of proteases and chitinases^15,16^ (our unpublished data). These multi-gene families probably represent a resource for the identification of lytic enzymes that have the potential to generate novel antifungal compounds.

## Methods

### Strains

Wild-type *Saccharomycopsis schoenii* (CBS 7425, CBS-KNAW collection, Utrecht, Netherlands) was provided by Marc-André Lachance. *Schizosaccharomyces pombe* UoA324, a derivative of FY15112 from NBRP yeast and SO2427 from Snezhana Oliferenko (King’s College London). Clinical isolates of the following *C. auris* and other *Candida* strains were used; *C.auris* (NCPF8980#9), *C. auris* (NCPF8985#20) and *C. auris* (NCPF13005#95) provided by Liz Johnson (PHE Bristol) and *C. auris* (B8441) provided by Shawn Lockhart (CDC Atlanta), *Candida albicans* (UC820) provided by Mihai Netea (Radboudumc, Nijmegen), *Candida glabrata* (BG2) provided by Brendan P. Cormack (Johns Hopkins University, Baltimore, MD), and *Candida parapsilosis* (AM2017/001), *Candida lusitaniae* (AM2017/006) and *Candida tropicalis* (AM2017/004) provided by Donna MacCallum (University of Aberdeen).

### Media and Growth conditions

Cells were cultured to log phase in YPD media (10 g/L yeast extract, 20 g/L Bacto peptone, 20 g/L glucose), 30 °C, rotating. Cells were washed and stained with Calcofluor White (10 µg/mL) and propidium iodide (1 µg/mL). *S. schoenii* was mixed with prey cells at roughly 5*10^7^ cells/mL each, in Synthetic Defined (SD) media (6.7 g/L YNB w/o amino acids with ammonium sulfate, 20 g/L glucose). Prior to imaging, cells were seeded on pads with SD media solidified with 1% agarose.

### Microscopy

Imaging was performed using the PerkinElmer UltraVIEW VoX Spinning Disk Confocal Microscope controlled by Volocity software. Images for movies were captured 2–4 times/min for up to 2 h, using the Nikon Perfect Focus System to autofocus. For kill curve analyses, three frames were captured every hour per species and time point. FIJI/ImageJ^17^ was used for image processing and analysis. Drift in movies frames was corrected using the macro NMS fixTranslation v1 and the plugin Image Stabiliser. For kill curve analyses, individual cells were counted using the Cell counter plugin.

For TEM images, *S. schoenii* and *C. auris* cells were separately pre-cultured to log phase in YPD media, then washed and mixed together at equal ratios. A total 1*10^8^ cells were seeded on SD media solidified with 2% agarose. After 1 h of co-culture, cells were scraped off, washed and pelleted. High Pressure Freezing was carried out using a Leica EM PACT 2 (Leica Microsystems, Milton Keynes, UK) and samples were freeze substituted in a Leica AFS 2. Freeze substitution was carried out using the following program: −95 °C to −90 °C for 30 h with 2% OsO_4_ in acetone, −90 °C for 10 h with 2% OsO_4_ in acetone, −90 °C to −30 °C for 8 h with 2% OsO_4_ in acetone, −30 °C to −10 °C for 1 h with acetone, −10 °C to 4 °C for 1 h in acetone, 4 °C to 20 °C for 1 h in acetone. Samples were then removed and placed in 10% Spurr’s (TAAB, UK): acetone for 72 h, followed by 30% Spurr’s overnight, 50% Spurr’s for 8 h, 70% Spurr’s overnight, 90% Spurr’s for 8 h and embedded in Spurr’s resin at 60 °C for at least 24 h. Ultrathin sections were cut to 90 µm using a diamond knife (Diatome Ltd, Switzerland) onto copper grids (TAAB, UK) using a Leica UC6 and were contrast stained with uranyl acetate and lead citrate in a Leica AC20. Samples were imaged on a JEM 1400 plus (JEOL UK) Transmission Electron Microscope and captured using an AMT UltraVUE camera (AMT, USA). All relevant data are available from the authors.

## Electronic supplementary material

Supplementary Information

Movie 1

Movie 2

Movie 3
