# Supplementary material for: The mycoparasitic yeast Saccharomycopsis schoenii predates and kills multi-drug resistant Candida auris
Source: Sci Rep. 2018 Oct 8;8:14959. doi: 10.1038/s41598-018-33199-z (PMC6175896; doi:10.1038/s41598-018-33199-z)
Supplement: Supplementary file 1 — Supplementary Information [file 41598_2018_33199_MOESM1_ESM.docx]

Junker *et al.,* **SREP-18-19093A**
**The mycoparasitic yeast *Saccharomycopsis schoenii predates* and kills multi-drug resistant *Candida auris***

**Klara Junker^1^, Gustavo Bravo Ruiz^2^, Alexander Lorenz^2^, Louise Walker^2^, Neil A.R. Gow^2*^ and Jürgen Wendland^1,3*^**

**^1^**Carlsberg Research Laboratory, Yeast & Fermentation, DK-1799 Copenhagen V, Denmark

^2^The Institute of Medical Sciences (IMS), MRC Centre for Medical Mycology at the University of Aberdeen, School of Medicine, Medical Sciences & Nutrition, University of Aberdeen, Foresterhill, Aberdeen, AB25 2ZD, United Kingdom

**^3^** Vrije Universiteit Brussel, Functional Yeast Genomics, BE-1050 Brussels, Belgium

**Running title: Mycoparasitism of *Saccharomycopsis schoenii* on *Candida auris***

* Corresponding authors
**Prof. Dr. Jürgen Wendland Prof. Dr. Neil Gow
Vrije Universiteit Brussels** The Institute of Medical Sciences (IMS)

**Research Group Microbiology** MRC Centre for Medical Mycology

**Functional Yeast Genomics** University of Aberdeen

**Pleinlaan 2** Foresterhill, Aberdeen, AB25 2ZD

**BE-1050 Brussels, Belgium** United Kingdom
Tel.: +45-3327-5230 +44-1224-437483

Fax: +45-3327-4708
email: [jurgen.wendland@vub.be](mailto:jurgen.wendland@vub.be) n.gow@abdn.ac.uk

**Supplementary Information**


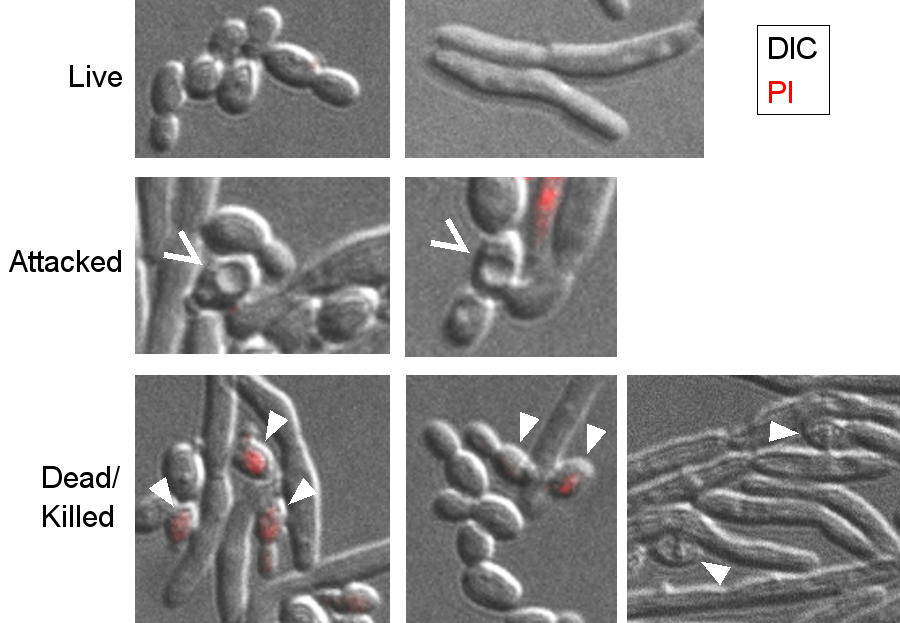


**Supplementary Figure 1:**

**Morphology and Propidium Iodide (PI) guided viability score.** We counted and scored prey cells and *S. schoenii* cells based on morphology and/or PI stain (red). Top row: Live cells were large with smooth morphology and no PI stain, *C. auris* to the left, *S. schoenii* to the right. Middle row: Attacked prey cells (Λ) were vacuolarized and in physical contact with *S. schoenii* cells. Bottom row: Dead prey cells (Δ) were stained by PI (left) and/or were shrunken in size (middle) or flattened (right).


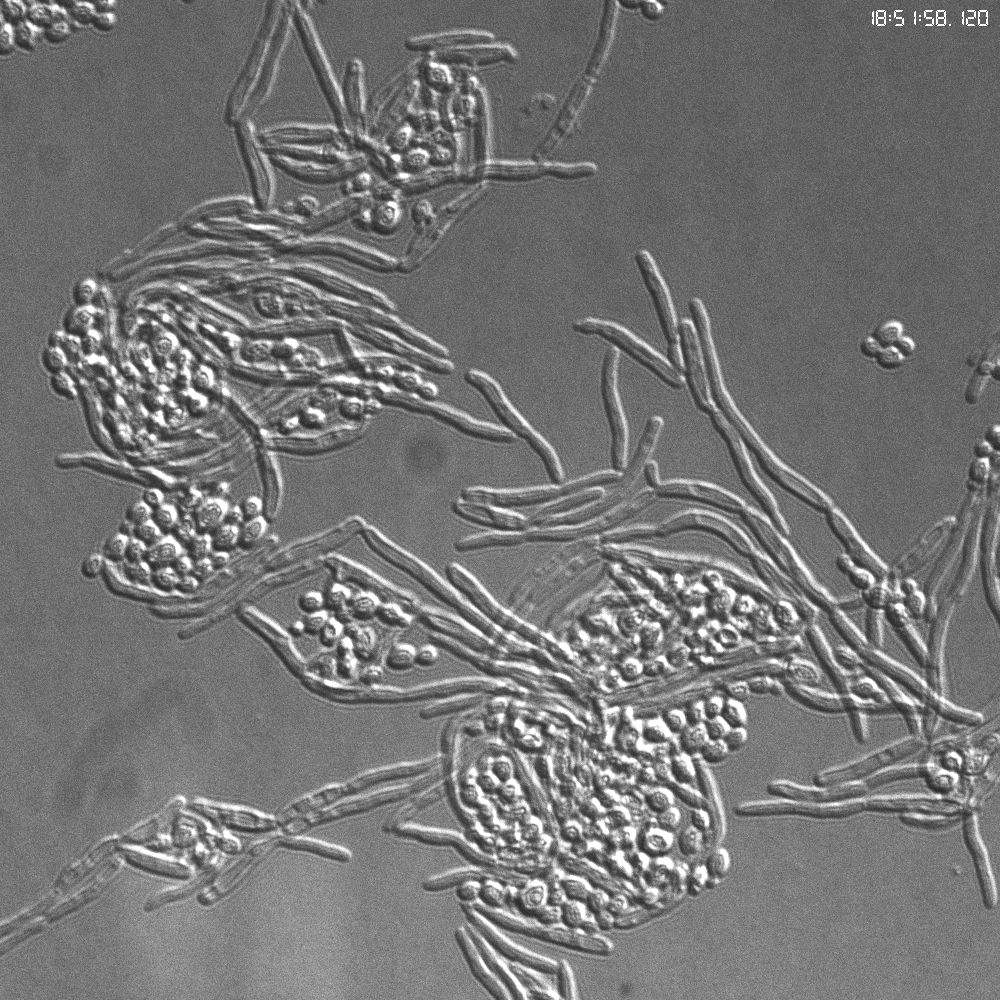


**Supplementary Figure 2**

**Example of overgrowth of *S. schoenii* cells and *C. auris (NCPF8980#9)* cells after 6 h of co-culture.** Quantitative observations were done hourly for up to 6 h, after which point individual cells could no longer be distinguished.

**Supplementary Table 1** Details of yeast strains used in this article.

| **Species name** | **Collection ID** | **Source** | **Genetic features** | **Clinical Features** |
| --- | --- | --- | --- | --- |
| *Saccharomycopsis schoenii* | CBS 7425 | CBS-KNAW collection (Utrecht, Netherlands) | Wild type |  |
| *Saccharomyces cerevisiae* | BY4741 | EUROSCARF | *his3*Δ*1;leu2*Δ*0;met15*Δ*0; ura3*Δ*0* |  |
| *Candida auris* | NCPF8980#9 | Elizabeth Johnson (PHE Bristol) | South African clade | • Isolated from blood. |
|  |  |  |  | • Resistant to Fluconazole |
| *Candida auris* | NCPF8985#20 | Elizabeth Johnson (PHE Bristol) | South Asian clade (India) | • Isolated from wound. |
|  |  |  |  | • Resistant to Fluconazole, Isovuonazole, Posaconazole, Voriconazole, Flucytosine, Anidulafungin |
| *Candida auris* | NCPF13005#95 | Elizabeth Johnson (PHE Bristol) | South African clade | • Isolated from urine. |
|  |  |  |  | • Resistant to Fluconazole, Voriconazole, Anidulafungin, AmBisome (possible low level) |
| *Candida auris* | B8441 | Shawn Lockhart (CDC Atlanta) | South Asian clade (Pakistan) | • Isolated from blood. |
|  |  | <https://doi.org/10.1093/cid/ciw691> |  | • Sensitive to antifungals |
| *Candida glabrata* | BG2 | Brendan P. Cormack (Johns Hopkins University, Baltimore, MD) |  |  |
|  |  | <http://science.sciencemag.org/content/285/5427/578> |  |  |
| *Candida albicans* | UC820 | Mihai Netea (Radboudumc, Nijmegen) |  |  |
|  |  | <http://jb.asm.org/content/98/3/996> |  |  |
| *Candida parapsilosis* | AM2017/001 | Donna MacCallum (University of Aberdeen) | Wild type |  |
| *Candida lusitaniae* | AM2017/006 | Donna MacCallum (University of Aberdgeen) |  |  |
| *Candida tropicalis* | AM2017/004 | Donna MacCallum (University of Aberdeen) |  |  |
| *Schizosaccaharomyces pombe* | UoA324 | Derivative of: FY15112, NBRP yeast and SO2427, Snezhana Oliferenko (King’s College London) | *h^-smt0^ hht1+::GFP-HA-kanMX uch2+::mCHERRY-ura4+ ura4-D18 leu1-32 his3-D1 arg3-D4* |  |

**Supplementary Table 2**

Number of *S. schoenii* cell and prey cells counted and viability scored at each time point.

|  |  | ***Prey cells*** | | | ***S. schoenii cells*** | |
| --- | --- | --- | --- | --- | --- | --- |
| **Prey species** | **Hour** | **Live** | **Attacked** | **Dead** | **Live** | **Dead** |
| *S. cerevisiae* | 0 | 124 | 0 | 1 | 94 | 2 |
|  | 1 | 181 | 29 | 1 | 199 | 2 |
|  | 2 | 54 | 16 | 113 | 232 | 12 |
|  | 3 | 22 | 9 | 148 | 315 | 8 |
|  | 4 | 24 | 1 | 128 | 317 | 4 |
|  | 5 | 5 | 2 | 102 | 415 | 11 |
|  | 6 | 9 | 4 | 142 | 430 | 5 |
| *C. albicans* | 0 | 148 | 1 | 0 | 131 | 5 |
|  | 1 | 186 | 20 | 65 | 194 | 7 |
|  | 2 | 172 | 10 | 99 | 157 | 0 |
|  | 3 | 123 | 8 | 108 | 174 | 0 |
|  | 4 | 83 | 10 | 149 | 230 | 0 |
|  | 5 | 79 | 10 | 144 | 220 | 4 |
|  | 6 | 63 | 5 | 103 | 198 | 0 |
| *S. pombe* | 0 | 52 | 1 | 3 | 107 | 2 |
|  | 1 | 64 | 4 | 1 | 126 | 1 |
|  | 2 | 72 | 0 | 18 | 161 | 2 |
|  | 3 | 36 | 3 | 11 | 120 | 1 |
|  | 4 | 39 | 3 | 17 | 186 | 4 |
|  | 5 | 61 | 6 | 32 | 196 | 1 |
|  | 6 | 54 | 9 | 27 | 232 | 1 |
| *C. auris* | 0 | 152 | 1 | 0 | 89 | 4 |
| NCPF8980#9 | 1 | 302 | 0 | 0 | 166 | 8 |
|  | 2 | 361 | 16 | 31 | 282 | 1 |
|  | 3 | 351 | 38 | 60 | 327 | 4 |
|  | 4 | 391 | 20 | 83 | 272 | 4 |
|  | 5 | 417 | 35 | 99 | 315 | 4 |
|  | 6 | 144 | 16 | 205 | 490 | 8 |
| *C. auris* | 0 | 314 | 0 | 1 | 140 | 3 |
| NCPF8985#20 | 1 | 232 | 9 | 6 | 143 | 3 |
|  | 2 | 291 | 28 | 59 | 221 | 8 |
|  | 3 | 533 | 20 | 122 | 328 | 1 |
|  | 4 | 361 | 31 | 135 | 340 | 2 |
|  | 5 | 295 | 32 | 139 | 369 | 5 |
|  | 6 | 201 | 16 | 87 | 194 | 1 |
| *C. auris* | 0 | 195 | 2 | 1 | 60 | 2 |
| NCPF13005#95 | 1 | 329 | 6 | 3 | 232 | 1 |
|  | 2 | 339 | 15 | 26 | 287 | 3 |
|  | 3 | 303 | 22 | 38 | 302 | 2 |
|  | 4 | 281 | 49 | 105 | 354 | 3 |
|  | 5 | 324 | 54 | 164 | 399 | 2 |
|  | 6 | 250 | 54 | 205 | 427 | 10 |
| *C. auris* | 0 | 184 | 0 | 0 | 167 | 4 |
| B8441 | 1 | 253 | 16 | 4 | 253 | 9 |
|  | 2 | 256 | 22 | 66 | 287 | 6 |
|  | 3 | 200 | 28 | 114 | 274 | 8 |
|  | 4 | 153 | 23 | 178 | 448 | 13 |
|  | 5 | 152 | 13 | 148 | 421 | 4 |
|  | 6 | 81 | 10 | 148 | 502 | 8 |
| *C. glabrata* | 0 | 221 | 2 | 1 | 152 | 2 |
|  | 1 | 217 | 22 | 53 | 137 | 1 |
|  | 2 | 248 | 15 | 180 | 215 | 2 |
|  | 3 | 222 | 11 | 210 | 196 | 5 |
|  | 4 | 139 | 10 | 194 | 212 | 4 |
|  | 5 | 84 | 11 | 217 | 360 | 1 |
|  | 6 | 108 | 9 | 179 | 301 | 5 |
| *C. parapsilosis* | 0 | 110 | 0 | 0 | 94 | 0 |
|  | 1 | 151 | 23 | 29 | 165 | 0 |
|  | 2 | 169 | 18 | 77 | 192 | 0 |
|  | 3 | 162 | 23 | 120 | 199 | 0 |
|  | 4 | 144 | 21 | 128 | 234 | 3 |
|  | 5 | 149 | 16 | 163 | 370 | 2 |
|  | 6 | 114 | 14 | 176 | 365 | 2 |
| *C. lusitaniae* | 0 | 146 | 0 | 2 | 105 | 2 |
|  | 1 | 161 | 28 | 47 | 198 | 3 |
|  | 2 | 152 | 14 | 82 | 184 | 2 |
|  | 3 | 230 | 24 | 198 | 250 | 0 |
|  | 4 | 315 | 23 | 179 | 244 | 2 |
|  | 5 | 221 | 29 | 232 | 316 | 2 |
|  | 6 | 216 | 20 | 158 | 278 | 0 |
| *C. tropicalis* | 0 | 161 | 0 | 0 | 83 | 1 |
|  | 1 | 242 | 29 | 95 | 189 | 1 |
|  | 2 | 261 | 11 | 173 | 215 | 3 |
|  | 3 | 139 | 22 | 224 | 233 | 0 |
|  | 4 | 113 | 19 | 207 | 262 | 0 |
|  | 5 | 135 | 16 | 207 | 269 | 2 |
|  | 6 | 171 | 15 | 190 | 247 | 3 |

**
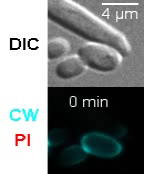
**

**Supplementary Movie 1 – Attached as “Supplementary Movie 1.mov”**

**Movie corresponding to Figure 1; *S. schoenii* attacks and kills *C. auris***. *S. schoenii* and *C. auris* NCPF8985#20 were stained with Calcoflour White (CW, cyan, bottom panel), a fluorescent dye that stains chitin rich cell walls and septa, and propidium iodide (PI, red, bottom panel), a fluorescent dye that stains nucleic acids of cells with a compromised cell membrane, i.e. dead or dying cells.

**
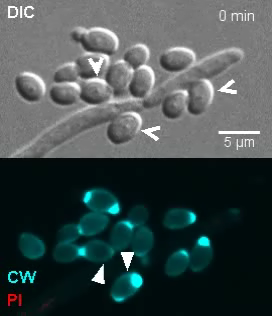
**

**Supplementary Movie 2 – Attached as “Supplementary Movie 2.mov”**

***S. schoenii* cells attacking three *C. auris* cells**. *S. schoenii* and *C. auris* NCPF8985#20 stained with Calcoflour White (CW, cyan in bottom panel) and propidium iodide (PI, red, bottom panel). Penetration pegs are visualized by appearance of CW staining at the site of penetration, at 20-30 min (Δ, lower panel). Prey cells collapse within minutes of the establishment of the penetration peg, at 25-40 min (Λ, upper panel). Weak PI staining is detected from 80-100 min.

**
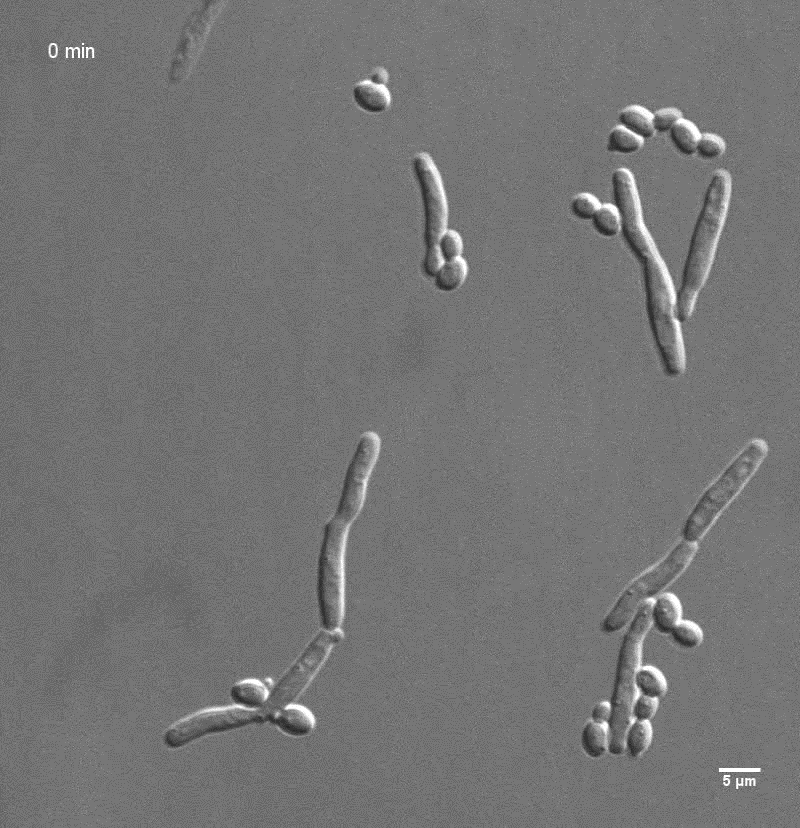
**

**Supplementary movie 3 – Attached as “Supplementary Movie 3.mov”**

**Several *S. schoenii* cells are sequentially attacking *C. auris* cells**. Arrows indicate attacked and killed *C. auris* NCPF8985#20 cells. Image acquisition (4/min) over 3 h.
